# A Dynamic View of ATP-coupled Functioning Cycle of Hsp90 N-terminal Domain

**DOI:** 10.1038/srep09542

**Published:** 2015-04-13

**Authors:** Huaqun Zhang, Chen Zhou, Wuyan Chen, Yechun Xu, Yanhong Shi, Yi Wen, Naixia Zhang

**Affiliations:** 1 Department of Analytical Chemistry, Shanghai Institute of Materia Medica, Chinese Academy of Sciences, Shanghai 201203, P. R. China; 2 CAS Key Laboratory of Receptor Research, Drug Discovery and Design Center, Shanghai Institute of Materia Medica, Chinese Academy of Sciences, Shanghai 201203, P. R. China; 3 State Key Laboratory of Drug Research, Shanghai Institute of Materia Medica, Chinese Academy of Sciences, Shanghai 201203, P. R. China

## Abstract

Heat-shock protein 90 (Hsp90) is one of the most important chaperones involved in multiple cellular processes. The chaperoning function of Hsp90 is intimately coupled to the ATPase activity presented by its N-terminal domain. However, the molecular mechanism for the ATP-dependent working cycle of Hsp90 is still not fully understood. In this study, we use NMR techniques to investigate the structural characteristics and dynamic behaviors of Hsp90 N-terminal domain in its free and AMPPCP (ATP analogue) or ADP-bound states. We demonstrated that although AMPPCP and ADP bind to almost the same region of Hsp90, significantly different effects on the dynamics behaviors of the key structural elements were observed. AMPPCP binding favors the formation of the active homodimer of Hsp90 by enhancing the slow-motion featured conformational exchanges of those residues (A117–A141) within the lid segment (A111–G135) and around region, while ADP binding keeps Hsp90 staying at the inactive state by increasing the conformational rigidity of the lid segment and around region. Based on our findings, a dynamic working model for the ATP-dependent functioning cycle of Hsp90 was proposed.

Heat-shock protein 90 (Hsp90) is one of the most important chaperones, which is involved in many cellular processes including cell cycle control, signal transduction and cell growth regulation^1^,^2^,^3^. Hsp90 achieves its function by facilitating maturation, stabilization, activation and intracellular sorting of more than 280 client proteins including oncogenic factors such as Raf-1, Akt, Src and Tert (http://www.picard.ch/downloads/Hsp90interactors.pdf). Besides, the cytosolic Hsp90α, an isoform of Hsp90 proteins encoded in humans, was proved to be stress-inducible and significantly up-regulated in certain types of cancer cells^4^,^5^,^6^. Therefore Hsp90 has a pivotal role in tumorigenesis and is serving as a promising target for anti-cancer drug development^7^,^8^,^9^,^10^.

The three-dimensional structures of Hsp90 from different species are very similar. Typically, Hsp90 consists of four structural domains: the highly conserved N-terminal domain with ATPase activity and co-chaperone recognition function; the middle domain which carries client protein and co-chaperone recognition function; the "charged linker" that connects the N-terminus with the middle domain; and the C-terminal domain which mediates formation of Hsp90 homodimer. All of the structural domains play key roles in the normal function of Hsp90^11^,^12^,^13^. In the resting state, through the dimerization of its C-terminal domain, Hsp90 forms a homodimer which is defined as the open conformation. Upon ATP binding the N-terminal domain undergoes significant conformational changes and comes in contact to form an active closed conformation. After Hsp90 finishes its chaperoning tasks of assisting the proper folding, stabilization and activation of client proteins under the active state, ATP molecule is hydrolyzed to ADP which then dissociates from Hsp90 and directs the protein back to the resting state. The functioning cycle of Hsp90 is regulated by more than 20 co-chaperones including p23, Cdc37 and Aha1, some of which play the tuning roles by either activating or inhibiting the ATPase activity of Hsp90 through interacting with the protein in different states^14^,^15^,^16^.

The chaperoning function of Hsp90 is intimately coupled to its ATPase activity. However, the dynamic molecular mechanism for the ATP-coupled functioning cycle of Hsp90 is still not fully understood. More importantly, the controversial data obtained by different approaches definitely cause even more confusions to the elucidation of the dynamic process. For example, the crystal structure of Hsp90 N-terminal domain alone showed no significant structural differences from those of its ATP analogue-bound states^17^, whereas the NMR experiment done by Karagöz *et al.* showed that ATP did induce the conformational changes restricted to the N-terminal domain of the human full-length Hsp90β in solution^18^. These results raised a basic scientific question of whether it is necessary and sufficient for ATP binding to induce the formation of the active conformation of Hsp90 N-terminal domain. To answer this question and achieve a dynamic view of the ATP-coupled functioning cycle of Hsp90 N-terminal domain, we use NMR techniques to investigate the structural characteristics and dynamic behaviors of the N-terminal domain in the absence and presence of either the non-hydrolysable AMPPCP (ATP analogue) or ADP in this study.

## Results and Discussion

### Backbone resonance assignments of Hsp90 N-terminal domain in its free and AMPPCP- or ADP-bound states

**Figure 1:**
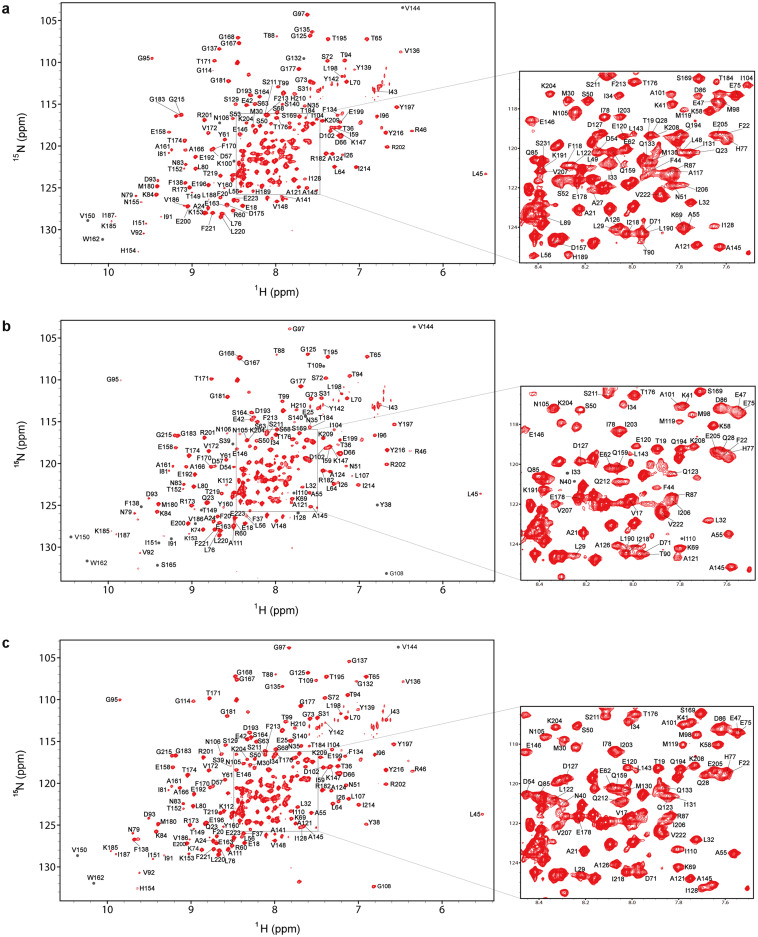
[^1^H, ^15^N] TROSY-HSQC spectra of the human Hsp90 N-terminal domain in its (a) free, (b) AMPPCP- and (c) ADP-bound states. Backbone amide resonance assignments are labeled with one-letter amino acid code and sequence number. Grey dots indicate resonances which are invisible at the current contour level. Regions with cross-peaks partially overlapped are zoomed in.

The backbone resonance assignments for the N-terminal domain of human Hsp90 in its free state have been reported^19^,^20^. These existing assignments are transferred onto our spectra and checked by 3D triple-resonance experiments. 183 out of 223 non-proline residues are finally identified (Fig. 1a, Supplementary Table S1 online). However, although the apo state backbone assignments of Hsp90 N-terminal domain have been available for some time, the function-relevant bound-state assignments are still absent. It could be partially attributed to the fact that the binding between Hsp90 and the nucleotides is in the slow exchange regime of NMR time scale, which makes the affected resonances very difficult to be followed from their free to bound states with increasing amounts of nucleotides. Therefore, a classical strategy, in which a series of 3D triple-resonance spectra are required, is employed to obtain the backbone chemical shift assignments for the AMPPCP/ADP-bound states of Hsp90 N-terminal domain. In total, 170 non-proline residues were identified for the triple-labeled Hsp90 bound with AMPPCP, while 178 non-proline residues were identified for the Hsp90:ADP complex (Fig. 1b and 1c, Supplementary Table S1 online). Dehner et al. analyzed the backbone resonances for the 25 kD N-terminal ATPase domain of yeast Hsp90 complexed with AMPPNP or ADP using a different approach based on the minimum deviation principle in chemical shift perturbations^21^. The advantage of this approach is economic and time-saving, but the accuracies of chemical shift assignments for individual residues, especially for those probably involved in conformational changes, might be out of reaching. Our accurate assignments for human Hsp90 N-terminal domain complexed with AMPPCP/ADP provide a solid base for structural characterization of Hsp90 upon nucleotides binding, and may be of benefit to a variety of researchers working on functional study and inhibitor discovery of Hsp90.

### AMPPCP and ADP bind to almost the same region of Hsp90 N-terminal domain but show different effects on the local conformation of its lid segment

**Figure 2:**
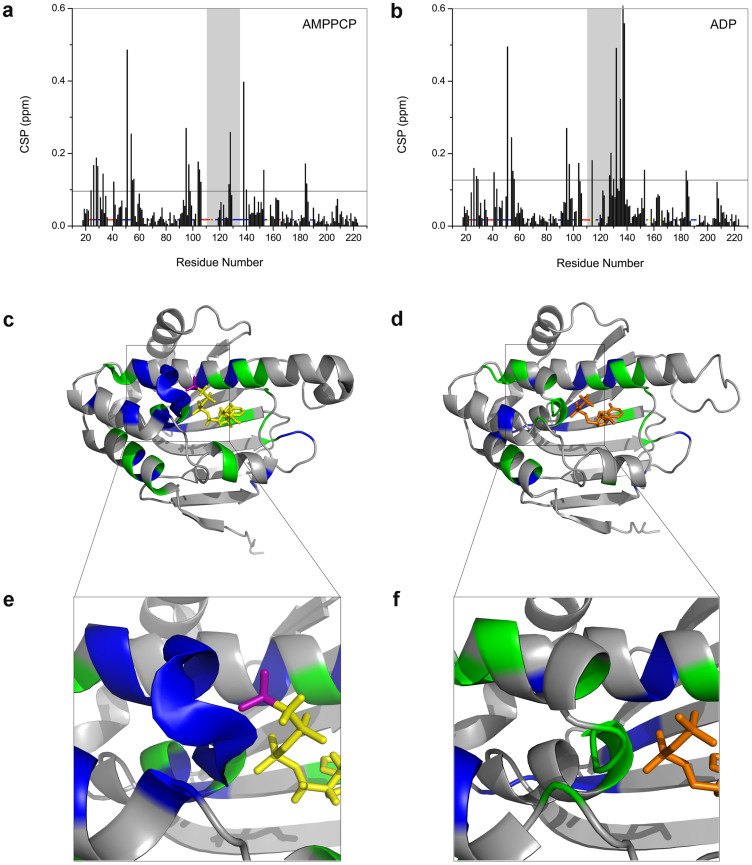
AMPPCP and ADP bind to almost the same region of Hsp90 N-terminal domain but show different effects on the local conformation of its lid segment. (a, b) Amide chemical shift perturbation (CSP) analysis reveals the residues involved in binding. Resonances assigned in the AMPPCP/ADP-bound states but not in the apo form (red dots), resonances unassigned in all three states (yellow dots), resonances attenuated upon AMPPCP/ADP binding (blue dots) and prolines in the sequence (black dots) are indicated. Residues with CSP values greater than 0.6 are marked X. The mean + SD value is indicated by a solid black line. The lid segment (residues A111–G135) is highlighted in grey. (c, d) Residues with resonance signals attenuated (blue) or CSP values above mean + SD (green) upon AMPPCP or ADP binding are mapped on the structures of Hsp90 (PDB IDs: 3T10 and 1BYQ). AMPPCP and ADP are indicated in yellow and orange sticks, respectively. The γ-phosphate in AMPPCP is highlighted in purple. (e, f) Zoomed-in views from (c) and (d).

The chemical shift value that an atom resonates at is sensitive to its chemical environment^22^. Chemical shift perturbation (CSP) analysis of target protein upon titration of its binding partners (ligands) therefore serves as a powerful method for identifying protein residues at contact surfaces or revealing structural changes of target protein induced by ligand binding. In our study, significant chemical shift perturbations upon the addition of AMPPCP/ADP were observed for a subset of amide resonances of Hsp90 N-terminal domain (see Supplementary Fig. S1 online), and the affected residues for AMPPCP/ADP binding were quite similar and shifted in almost the same directions to a comparable extent (see Supplementary Fig. S1 online), suggesting that AMPPCP and ADP bind to almost the same region of Hsp90 N-terminal domain. We then calculated the CSP values for each residue (Fig. 2a and 2b) and mapped them onto the 3D cartoon structures of Hsp90 N-terminal domain (Fig. 2c and 2d). Our results showed that the significantly affected residues mainly locate around the nucleotide-binding pocket, including the fragment A111–G135 which is called “lid” segment and involved in the formation of the active closed conformation of Hsp90^23^,^24^.

**Figure 3:**
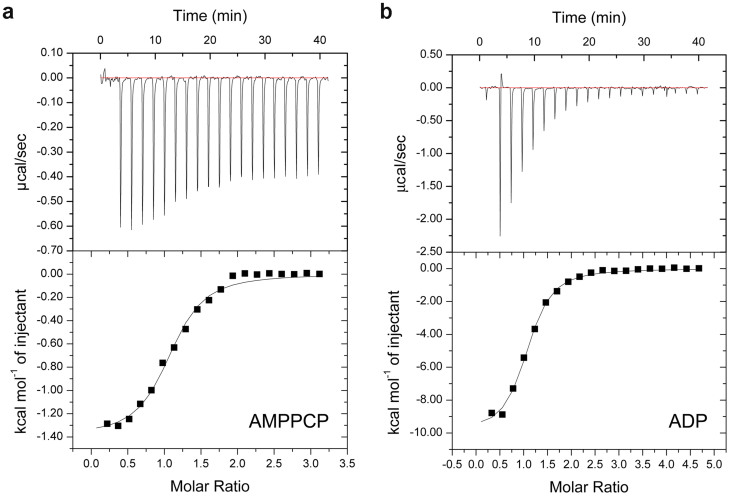
Isothermal titration calorimetry experiments were applied to determine the thermodynamic parameters for the binding of (a) AMPPCP or (b) ADP to Hsp90 N-terminal domain. The fitting thermodynamic parameters are summarized in Table 1.

Noticeably, different from the ADP-bound state, the amide resonances of ~10 amino acid residues locating in the lid segment or nearby, including M130–G137, Y139 and A141, are entirely absent from the recorded spectra of the AMPPCP-bound Hsp90 (Fig. 2e and 2f, Supplementary Table S1 online). The absence of these resonances suggested that in the nucleotide binding region of Hsp90, those residues, spatially close to the γ-phosphate group of AMPPCP, underwent conformational exchange in the millisecond time scale (as the data was acquired within 150 ms) when the protein was in AMPPCP-bound state. Besides, consistent with the above-mentioned NMR data, significantly different contributions of entropy change to Gibbs free energy change were observed in the isothermal titration calorimetry experiments for Hsp90:AMPPCP and Hsp90:ADP systems (Fig. 3 and Table 1), suggesting the AMPPCP binding and the ADP binding cause different effects on the local conformations of Hsp90. Since it has been revealed by the reported crystal structures of Hsp90 in their ATP analogue-bound states^17^,^24^,^25^ that the γ-phosphate group of ATP analogues are solvent-accessible, we cannot exclude the possibility that the solvation change of γ-phosphate group of AMPPCP also contributes to the entropy increase when it binds to Hsp90. In the meantime, the conformational variation of the γ-phosphate group itself in AMPPCP may also be a factor impacting on the different entropy change from that of ADP binding. Overall, we believe that the molecular recognition and binding of lid segment or nearby with AMPPCP is likely to experience a more complicated conformation-selected or conformation-induced process than that with ADP.

In order to confirm what we have observed from the binding of AMPPCP, we investigated the interaction between Hsp90 N-terminal domain and another widely-used ATP analogue AMPPNP. It was revealed by the NMR titration data that the NMR resonances of Hsp90 underwent significant chemical shift perturbations with the addition of AMPPNP (see Supplementary Fig. S1 online). Besides, the overall perturbation pattern of affected residues in Hsp90 N-terminal domain is almost the same as that induced by the AMPPCP binding (see Supplementary Fig. S1 online). In a previously published work, Karagoz et al. reported that no significant differences were observed in the NMR spectra of AMPPNP and ATP-loaded Hsp90, which indicates that both of them represent the ATP-bound state of Hsp90^18^. So we can extend the conclusion a bit more: the binding modes of Hsp90 with AMPPNP, AMPPCP and ATP are all identical. Consistently, the entropy change featured with a positive value was also observed in the isothermal titration calorimetry experiment for Hsp90:AMPPNP system (Table 1, Supplementary Fig. S2 online). From the NMR and ITC data, we can exclude the possibility that the observed effects described in this paper are just results from the specific ATP analogue AMPPCP.

In addition, previous studies have suggested that the binding affinity of ADP is higher than that of other ATP analogues to a certain extent^26^,^27^,^28^,^29^. Our ITC data shows that the binding affinities of AMPPNP and AMPPCP to Hsp90 N-terminal domain are at the same order of magnitude (Table 1). In comparison with ADP, the binding affinities of AMPPNP and AMPPCP to Hsp90 are slightly weaker or comparable (Table 1). The observed differences in absolute K_d_ values are possibly due to experimental condition (temperature, pH and ion strength) variations and the different constructs or protein sequences of Hsp90 applied.

### The conformational exchanges in μs-ms time scale of the mobile lid segment are promoted by the AMPPCP binding and inhibited with the addition of ADP

To probe the internal dynamics of the N-terminal domain of Hsp90 in its free and AMPPCP- or ADP-bound states, we measured ^15^N longitudinal (R_1_), transverse (R_2_) relaxation rates and heteronuclear ^1^H-^15^N NOEs (XNOE) (see Supplementary Fig. S3 online). In the preliminary analysis of above-mentioned original relaxation data, the commonly-used model-free approach^30^,^31^ was applied. However, the model-free analysis based on the atomic coordinates of available crystal structures of Hsp90 failed (data not shown) because the solution structures of Hsp90 in its free and AMPPCP- or ADP-bound states (see Supplementary Fig. S4 online) are not fully compatible with those reported crystal structures. We then employed the reduced spectral density mapping approach, which can be used to elucidate the internal dynamics of amide bond without any assumption for a specific molecular model, to interpret the relaxation data. The spectral density functions J(0), J(ω_N_) and J(0.87ω_H_) link to internal motions at low, intermediate and high frequencies, respectively. In particular, J(0) is sensitive to both slow (μs-ms) and fast (ps-ns) motions. Slow motions lead to an increase while fast motions cause a decrease in J(0) values^32^. Since protein conformational exchanges usually occur in the slow time scale, we then can identify those residues with potential conformational exchanges by comparing J(0) values.

**Figure 4:**
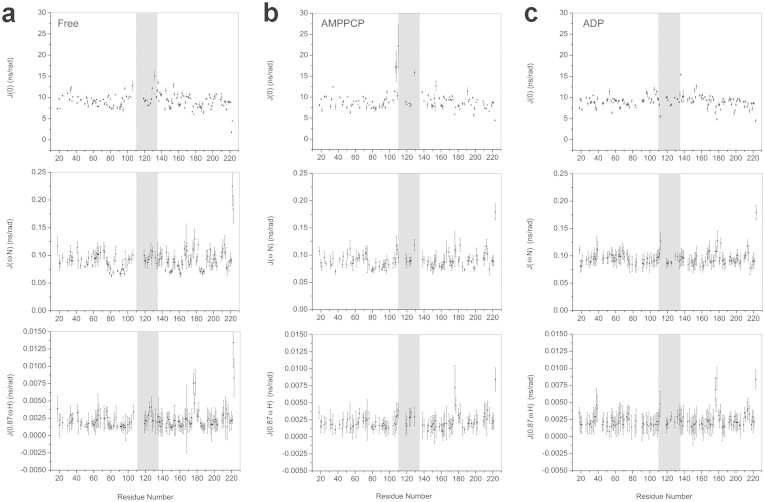
Reduced spectral density functions of the Hsp90 N-terminal domain in its (a) free, (b) AMPPCP- and (c) ADP-bound states. The lid segment (residues A111–G135) is highlighted in grey.

The average J(0) value for residues L107-A141 locating in the lid segment and around of Hsp90 N-terminal domain is 10.36 ns/rad (Table 2), which is higher than the mean value of 9.2 ns/rad for all residues of the apo protein (Fig. 4). These data suggested that the lid segment and around region of Hsp90 underwent conformational exchange in the μs-ms time scale. Besides, consistent with the relaxation data, most of the amide resonances from residues L107-K116 are entirely absent from the recorded spectra of Hsp90 in its free state (see Supplementary Table S1 online), indicating that these residues underwent conformational exchange in the ms time scale. Thus it is reasonable to consider that multiple conformations may exist around the lid segment of Hsp90 N-terminal domain in its free state. Indeed, in the absence of ATP, a considerably heterogeneous conformational ensemble is formed by human full-length Hsp90^20^. It has been also reported that multiple conformations for bacterial Hsp90 homolog HtpG coexist in equilibrium under physiologically relevant conditions^33^.

**Figure 5:**
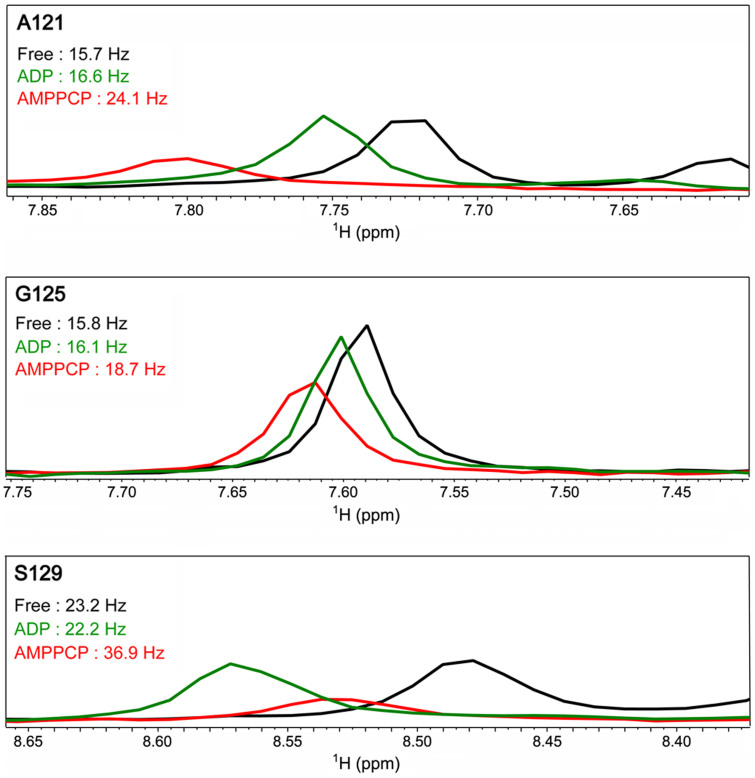
Line shape visualization of representative residues of Hsp90 N-terminal domain in its free (black), AMPPCP- (red) and ADP-bound states (green). The linewidths at half-height of NMR signals for representative residues are labeled.

Upon AMPPCP binding, the amide resonances of thirteen residues within the lid segment and around region (A117, F118, L122, M130, I131, G132, Q133, F134, G135, V136, G137, Y139 and A141) were severely broadened and missing from the ^1^H-^15^N HSQC spectrum of Hsp90 N-terminal domain (Fig. 1b, Supplementary Table S1 online). A possible reason for this could be that AMPPCP binding promotes the conformational exchanges of the fragment spanning A117–A141 in the ms time scale. Consistent with this hypothesis, observable residues within this region exhibit larger linewidths with the addition of AMPPCP (Fig. 5). In the meantime, conformational exchanges featured by motions in the μs-ms time scale were observed for the N-terminal six residues of the lid segment and around region (L107–K112) with the addition of AMPPCP. With the AMPPCP binding, the average J(0) value for residues L107–K112 of Hsp90 reached a high number of 16.73 ns/rad (Fig. 4, Table 2), and the intensities of amide resonances of these residues are quite small (see Supplementary Fig. S5 online). Additionally, the NMR assignment data showed that without the nucleotide binding the fragment spanning L107–K112 of Hsp90 underwent conformational exchanges in the ms time scale (Fig. 1a, Supplementary Table S1 online), so it is proposed that the AMPPCP binding triggers a change in internal motions for residues L107–K112 from ms to μs-ms time scale. Generally, it can be concluded that the low frequency motion related dynamics of the C-terminal part of lid segment and nearby was significantly enhanced by the AMPPCP binding, and the low frequency motion feature of the N-terminal part of this region was also altered.

With the ADP binding, the linewidths of residues within the lid segment and around are either smaller or comparable to the linewidths of these residues of Hsp90 in its free state (Fig. 5 and Supplementary Fig. S5 online). These data suggested that the ADP binding likely down-regulates the low frequency motion of the lid segment and around region of Hsp90 N-terminal domain. The result was further confirmed by the relaxation data. The average J(0) value of residues L107–A141 of Hsp90 in its ADP-bound state is 9.74 ns/rad, which was lower than that of Hsp90 in its free state (Fig. 4, Table 2). In fact, in the ADP-bound state, the residue K112 of Hsp90 even showed a small J(0) value at ~5.5 ns/rad (Fig. 4), indicating fast dynamics in the ps-ns time scale. The ADP binding had a different effect on Hsp90 by restricting the slow internal motions and enhancing the conformational rigidity of the lid segment.

### A dynamic working model for the ATP-dependent functioning cycle of the Hsp90 N-terminal domain

**Figure 6:**
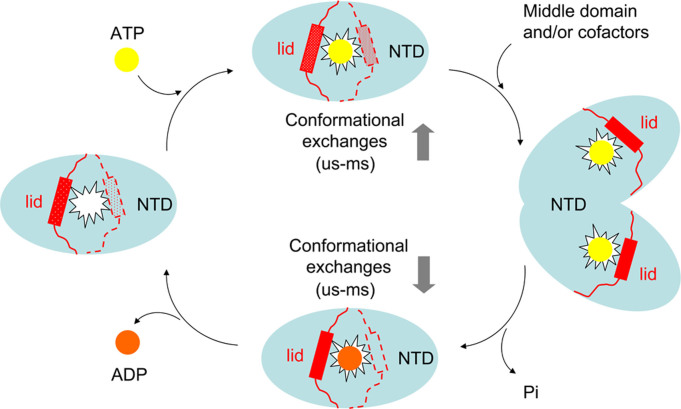
A proposed working model for the ATP-dependent functioning cycle of Hsp90. The N-terminal domain, abbreviated as NTD, is indicated by cyan ellipse. The nucleotide binding cavity is indicated as irregular star in the center of NTD. ATP and ADP molecules are indicated in yellow and brown circles, respectively. The phosphoric acid molecule is abbreviated as Pi. The lid segment (residues A111–G135) is indicated by a combination of rectangle and dots.

The lid segment was identified to play a crucial role in the formation of the active closed conformation of Hsp90^23^,^24^. Another structural element spanning E18–A21 was also reported to promote the dimerization of Hsp90 through swapping to interact with the partner chaperone protein^25^. The removal of the N-terminal 24 residues conferring flexibility specifically to the lid segment^34^ suggests that the nucleotide binding, the lid segment restructuring and the β-strand swapping are coupled. On the other hand, the non-hydrolysable AMPPCP is one of the common ATP analogues which are used to mimic the binding of ATP. As it has been indicated by the published work^18^ and the data presented in this paper, the binding modes of Hsp90 with AMPPCP and ATP are very close. Li et al. have also reported that the crystal structures of Hsp90N:ATP and Hsp90N:AMPPCP are identical and the two nucleotides are well overlapped^17^. So the binding effects of AMPPCP and ATP on the structures and dynamics of Hsp90 could be considered as equivalent. Using all our data and the previous findings a detailed working model for the ATP-dependent functioning cycle of Hsp90 was proposed (Fig. 6). The N-terminal domain of apo Hsp90 adopts a cluster of inactive conformations in its resting state in solution. However, the mobile lid segment spanning A111–G135 experiencing μs-ms internal motions entails the existence of transient conformations with higher energy at a low population. The ATP binding enhances the slow-motion-featured conformational exchanges of the residues spanning A117–A141 within the lid segment and region around and promotes the sparsely-populated active conformers, although the dominant conformations of ATP-bound Hsp90 are still inactive. With the coordination of the middle domain of Hsp90 and/or cofactors, the N-terminal domain forms an active homodimer via the swapping of the N-terminal β-strand spanning E18-A21. The diverse catalytic regulations of Hsp90 involved in different biological pathways are subsequently carried out by the activity-related high-energy dimer. After the accomplishment of the chaperoning tasks and the hydrolysis of ATP molecule, the dimeric N-terminal domain of Hsp90 dissociates to ADP-bound monomer state, which is favored in the inactive conformations. The internal dynamics of most of the residues within the lid segment is mainly restricted on the ps-ns time scale in the ADP-bound Hsp90. After ADP dissociates from the N-terminal domain, Hsp90 returns back to the resting state and completes its ATPase cycle. From this working model we came to the conclusion that it is necessary and sufficient for ATP binding to induce the formation of the active conformation of Hsp90 N-terminal domain; however, the middle domain and/or other cofactors are required to stabilize such an active conformation. It should be noted that the whole Hsp90 ATPase cycle is on the order of 0.1–1 hydrolysis event per minute, and the N-terminal dimerization is the rate-limiting step of the process^35^,^36^. Due to the high energy, it is very challenging to directly probe the ATP-bound active conformation of the dimeric conformer formed by Hsp90 N-terminal domain. The assistances of the middle domain and cofactors make the system even more complicated.

In summary, by using NMR techniques, the atomic dynamic picture of the ATP-dependent working process of Hsp90 was depicted. This work will be very helpful for a better understanding of the detailed molecular mechanism of the ATP-coupled functioning cycle of Hsp90. Our findings will also provide valuable information for the anti-Hsp90 drug development.

## Methods

### Preparation of NMR samples

The prokaryotic expression plasmid pET28a-Hsp90α (9–236) was generously given by Dr. Jianhua He (Shanghai Institute of Applied Physics, Chinese Academy of Sciences, Shanghai, China). His-tagged Hsp90α (9–236) was expressed in *Escherichia coli* and purified by using a combination of affinity chromatography and size exclusion chromatography on an FPLC system as described previously^17^. ^15^N, ^13^C, and ^2^H labeled samples were produced by growth in M9 minimal media with ^15^N labeled ammonium chloride, ^13^C labeled glucose and D_2_O used as the nitrogen, carbon and water sources, respectively.

### NMR spectroscopy

All NMR data for the free and AMPPCP/ADP-bound forms of the Hsp90 N-terminal domain, corresponding to the three conformational states in the ATPase cycle, were collected on either Bruker Avance III 600 or 800 MHz NMR spectrometers equipped with cryogenically cooled probe at 20°C.

Two dimensional [^1^H, ^15^N] HSQC titration experiments were recorded on 100% ^15^N and 70% ^2^H double labeled Hsp90α (9–236) with increasing molar ratios of AMPPCP, AMPPNP or ADP. For the line shape visualization purposes, three [ ^1^H, ^15^N] HSQC spectra were recorded on 100% ^15^N and 90% ^2^H double labeled Hsp90α (9–236) in its free state and with the addition of either 10-fold molar excess of AMPPCP or 5-fold molar excess of ADP. A series of 3D triple-resonance spectra including [^1^H, ^15^N, ^13^C] HNCA/HN(CO)CA pair, [^1^H, ^15^N, ^13^C] HNCO/HN(CA)CO pair, and [^1^H, ^15^N, ^13^C] HNCACB, which were acquired on 100% ^15^N, 100% ^13^C and 70% ^2^H labeled Hsp90α (9–236) in its free state and with the addition of either AMPPCP or ADP, were used to obtain chemical shift assignments. The concentrations of Hsp90α (9–236) for the 2D [^1^H, ^15^N] HSQC titrations and 3D triple-resonance experiments were 0.1–0.3 and 0.6 mM, respectively. The spectra were recorded in NMR buffer (20 mM Tris-HCl, 75 mM NaCl and 1 mM β-mercaptoethanol, at pH 7.4) containing Mg^2+^ ions at the AMPPCP-, AMPPNP- or ADP-to-MgCl_2_ ratio of 1:2.

For the backbone ^15^N relaxation experiments, the samples of 100% ^15^N and 90% ^2^H double labeled Hsp90α (9–236) in the presence of saturated molar excess of AMPPCP/ADP were used. The backbone ^15^N relaxation parameters, including longitudinal relaxation time (T_1_), transverse relaxation time (T_2_) and heteronuclear steady-state ^1^H-^15^N NOEs (XNOE), for free and AMPPCP/ADP-bound states of Hsp90α (9–236) were measured. All ^15^N-T_1_ and T_2_ relaxation experiments were carried out with a 3 s recycle delay between scans. Relaxation delays were set to 10*2, 100, 300, 500, 800, 1100, 1500, 2000, 2600, 3200 and 4000 ms for the T_1_ experiments and 7*2, 15, 22, 30, 37, 44, 52, 69, 89, 119 and 148 ms for the T_2_ experiments. The relaxation rates were determined by fitting the cross peak intensities to a single exponential function using nonlinear least-squares and the errors for the determined rate constants were assessed from Monte Carlo simulations. XNOE spectra were recorded in an interleave manner either with or without ^1^H saturation, and 8 s delays were used between scans to ensure the full recovery of ^15^N longitudinal magnetization. XNOE values were obtained by calculating the ratios of the peak intensities measured with and without proton saturation. Duplicate spectra were used to estimate experimental errors.

### NMR data analysis

NMR data processing and analysis was performed using programs NMRPipe^37^, CARA^38^ and Sparky (Goddard and Kneller, Sparky 3, University of California, San Francisco). Chemical shift perturbation values (Δδ_avg_) for ^15^N and ^1^H nuclei were derived from equation (1): 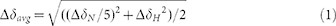
where Δδ_N_ and Δδ_H_ represent the chemical shift perturbation value of the amide nitrogen and proton, respectively. Chemical shift index analysis was done by using the program CSI-v2.0^39^,^40^, and the number −1, 0 or 1 was assigned to a particular residue whose observed chemical shift fell lower than, into the empirically determined range of, or higher than the corresponding reference value, respectively. Moreover, the internal motions of N-H bonds of Hsp90α (9–236) in free and AMPPCP/ADP-bound states were elucidated by using the spectral density mapping approach^41^,^42^,^43^. The reduced spectral density values were calculated as follows:
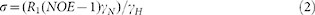

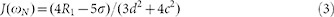

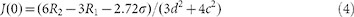

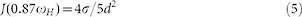
where *d* = *μ*_0_*hγ*_N_/*γ*_H_<*r*_NH_^−3^>/(8*π*^2^), *c* = ω_N_Δ*σ*/3^1/2^, *μ*_0_ is the permeability of the free space, *h* is the Planck's constant, *γ*_H_ and *γ*_N_ are the gyromagnetic ratios of ^2^H and ^15^N, respectively, *r*_NH_ is the N-H bond length, ω_H_ and ω_N_ are the Lamor frequencies of ^1^H and ^15^N, respectively, and Δ*σ* is the chemical shift anisotropy for ^15^N with Δ*σ* = *σ*_∥_ − *σ*_⊥_ = −160 ppm.

### Isothermal titration calorimetry measurements

All isothermal titration calorimetry measurements were performed on a MicroCal ITC200 instrument at 25°C. Protein samples were prepared at a concentration of 0.1 mM in assay buffer (20 mM Tris-HCl, 75 mM NaCl, 6 mM MgCl_2_ and 1 mM β-mercaptoethanol, pH 7.4) and added into a 200 μL cell. AMPPCP, AMPPNP or ADP powders were dissolved in the assay buffer and adjusted to pH 7.4. The AMPPCP, AMPPNP or ADP stocks at a final concentration of 1 mM were then transferred into a 40 μL syringe. 19 injections of 2 μL of ligands into the protein were carried out after an initial injection of 1 μL. A spacing time of 120 s was used for equilibration between injections. Data were subsequently analyzed with the Origin software package from MicroCal.

## Author Contributions

N.Z. and Y.W. designed the experiments and wrote the main manuscript text. H.Z. and C.Z. prepared figure 1, figure 2, figures 4–6 and table 2, and W.C. and Y.X. prepared figure 3 and table 1. Y.S. proofread the manuscript. All authors reviewed the manuscript.

## Supplementary Material

Supplementary Information supplementary information

## Figures and Tables

**Table 1:** Thermodynamic parameters of the Hsp90 N-terminal domain fitted from ITC experiments

Protein	Ligand	N	Kd (μM)	ΔH (10^3^ cal mol^−1^)	ΔS (cal mol^−1^ deg^−1^)
	AMPPCP	1.06 ± 0.02	3.80 ± 0.68	−1.40 ± 0.04	20.2
Hsp90N	AMPPNP	1.04 ± 0.03	7.93 ± 2.34	−3.12 ± 0.01	13.07
	ADP	1.03 ± 0.02	3.08 ± 0.34	−9.99 ± 0.22	−7.72

**Table 2:** The average J(0) values for residues in the lid segment or nearby of the Hsp90 N-terminal domain in different states

Fragment	J(0) (ns rad^−1^) (Free state)	J(0) (ns rad^−1^) (AMPPCP-bound state)	J(0) (ns rad^−1^) (ADP-bound state)
L107-A141	10.36	12.06	9.74
L107-K112	NA	16.73	9.19
S113-K116	NA	NA	NA
A117-S129	9.35	9.59	9.29
M130-A141	11.49	NA	10.59
