# Supplementary material for: A Dynamic View of ATP-coupled Functioning Cycle of Hsp90 N-terminal Domain
Source: Sci Rep. 2015 Apr 13;5:9542. doi: 10.1038/srep09542 (PMC4394755; doi:10.1038/srep09542)
Supplement: Supplementary Information [file srep09542-s1.pdf]

Supporting information

A Dynamic View of ATP-coupled Functioning Cycle of Hsp90 N-terminal Domain

Huaqun Zhang[1, #, ¶], Chen Zhou[1, #], Wuyan Chen[2],
Yechun Xu[2], Yanhong Shi[1], Yi Wen[1, *], Naixia Zhang[1, 3, **]

1 Department of Analytical Chemistry, Shanghai Institute of Materia Medica, Chinese Academy of Sciences, Shanghai 201203, P. R. China

2 CAS Key Laboratory of Receptor Research, Drug Discovery and Design Center, Shanghai Institute of Materia Medica, Chinese Academy of Sciences, Shanghai 201203, P. R. China

3 State Key Laboratory of Drug Research, Shanghai Institute of Materia Medica, Chinese Academy of Sciences, Shanghai 201203, P. R. China

¶ Current address: College of Arts and Science, Miami University, Oxford, OH 45056, USA

[#] These authors contributed equally to this work.

* Corresponding author, Tel: (86) 21-50806600 ext. 3121.

** Corresponding author, Tel: (86) 21-50806036.

E-mail address: wenyi1984112@simm.ac.cn (Y. Wen); nxzhang@mail.shcnc.ac.cn (N-X. Zhang).

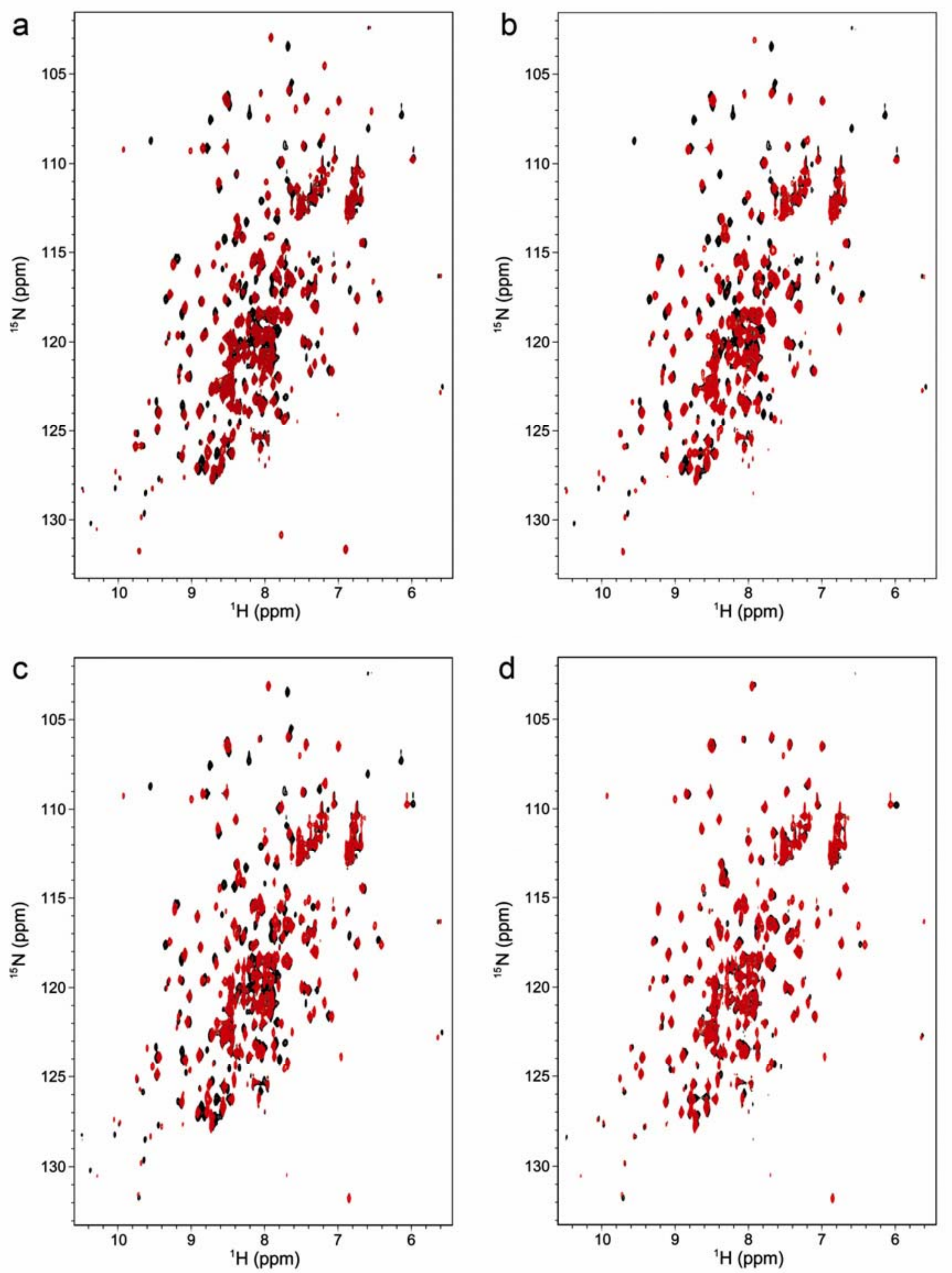

Figure S1. Superposition of [^{1}H, ^{15}N] HSQC spectra for the N-terminal domain of Hsp90 in different states. (a) Apo form (black) and ADP-bound state (red). (b) Apo form (black) and AMPPCP-bound state (red). (c) Apo form (black) and AMPPNP-bound state (red). (d) AMPPCP-bound state (black) and AMPPNP-bound state (red).

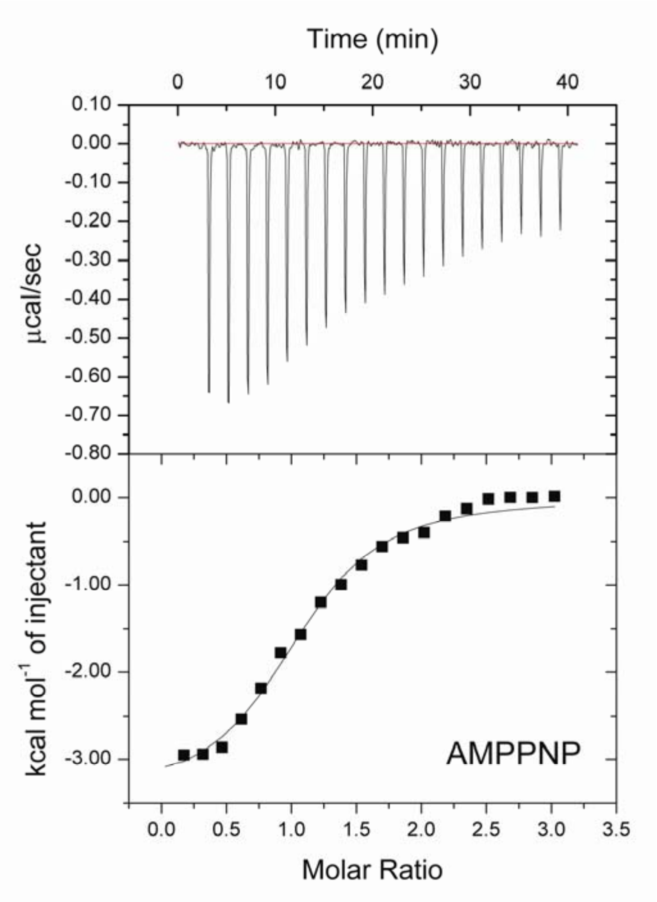

Figure S2. Isothermal titration calorimetry experiment was applied to determine the thermodynamic parameters for the binding of AMPPNP to Hsp90 N-terminal domain. The fitting thermodynamic parameters are summarized in Table 1.

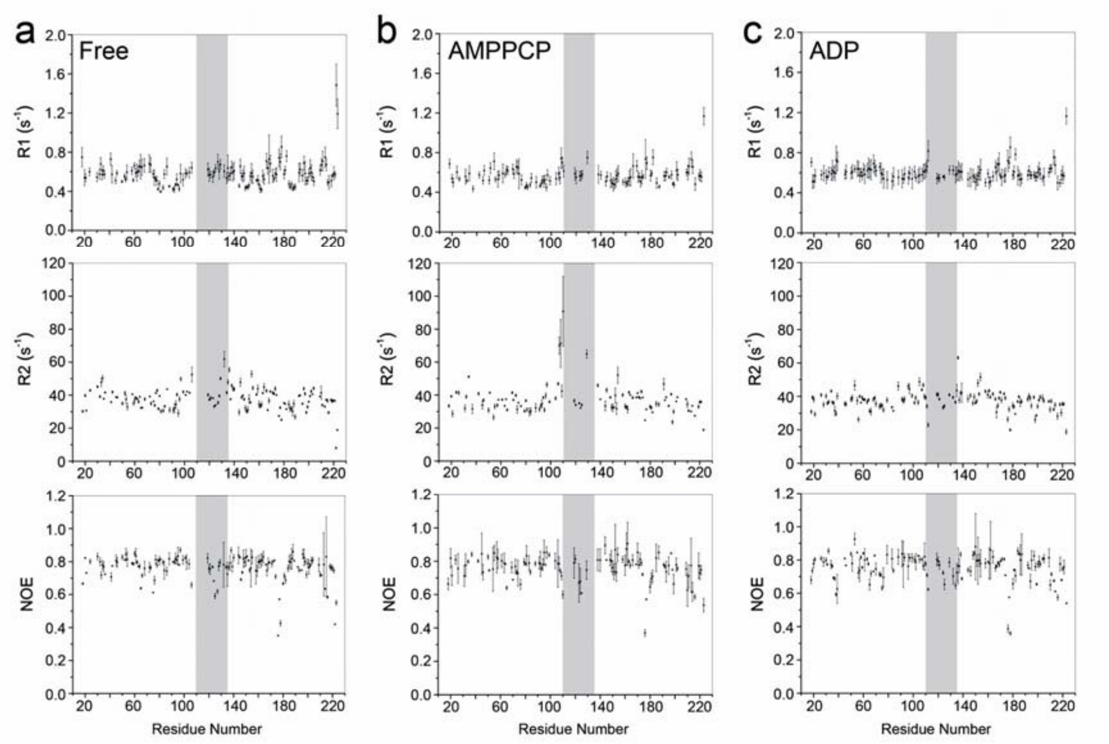

Figure S3. ^{15}N longitudinal relaxation rates R_1, transverse relaxation rates R_2 and {^{1}H}-^{15}N heteronuclear steady NOEs of the N-terminal domain of Hsp90 in its (a) free, (b) AMPPCP- and (c) ADP-bound states. The lid segment (residues A111-G135) is highlighted in grey.

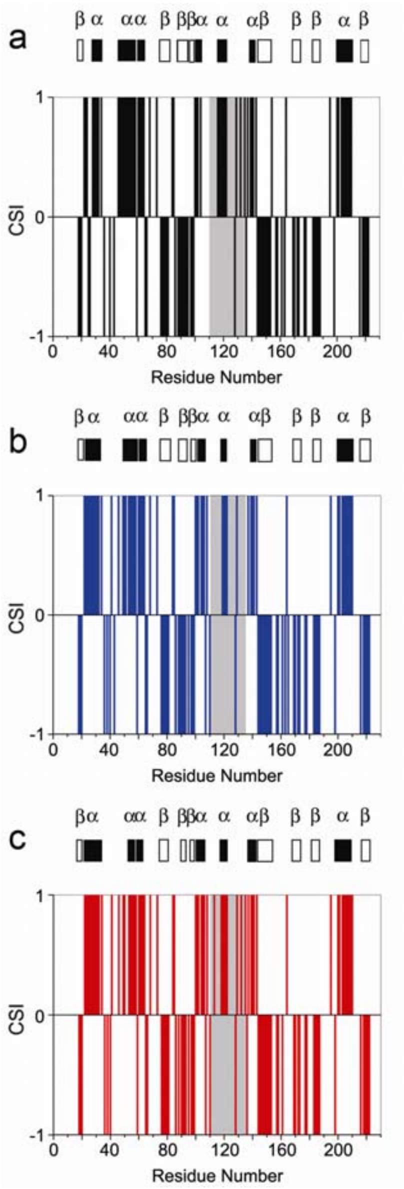

Figure S4. Chemical shift index (CSI) of the N-terminal domain of Hsp90 in its (a) free, (b) AMPPCP- and (c) ADP-bound states. The plot is derived from the consensus characterization of CA and CO. The lid segment (residues A111-G135) is highlighted in grey. The secondary structure elements are indicated on the top. Subtle differences between the solution structure of Hsp90 N-terminal domain and its crystal structure are revealed by the CSI data. In solution, residues I128-F134 locating in the lid segment do not form the α-helix element which is observed in the crystal structures for the free (PDB ID: 3T0H), AMPPCP- (PDB ID: 3T10) or ADP- bound state (PDB ID: 1BYQ) of Hsp90. Residues E192-L198 are identified unstructured by CSI while a long α-helix element extending from E192 to S211 is observed in all of the above mentioned three crystal structures. These differences strongly indicate that the existing crystal atomic coordinates are not suitable for the model-free analysis to disclose the dynamics behavior of Hsp90 in solution.

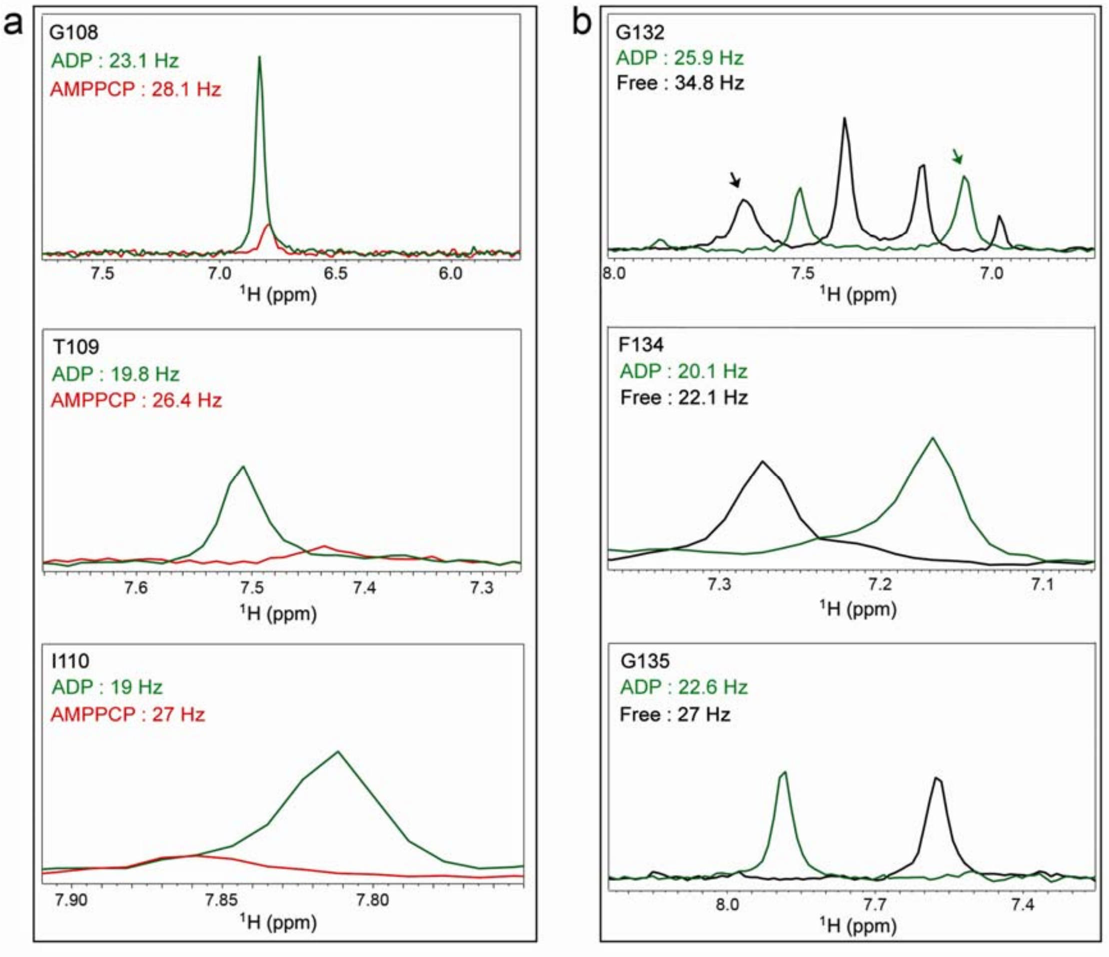

Figure S5. Line shape visualization for representative residues in (a) fragment L107-K112 or (b) fragment M130-A141 of the Hsp90 N-terminal domain in its free (black), AMPPCP- (red) and ADP-bound states (green). The linewidths at half-height of NMR signals for representative residues are labeled.

Table S1. ^{1}H and ^{15}N resonance assignments of the N-terminal domain of Hsp90 in its free and AMPPCP/ADP-bound states.

Hsp90N Sequence	Free		AMPPCP		ADP	
	δ ^{1}H	δ ^{15}N	δ ^{1}H	δ ^{15}N	δ ^{1}H	δ ^{15}N
V17			8.074	120.985	8.062	120.91
E18	8.47	126.258	8.447	126.263	8.438	126.322
T19	7.988	118.676	7.949	118.404	7.94	118.379
F20	8.719	125.268	8.751	125.455	8.746	125.479
A21	8.285	122.304	8.273	122.629	8.263	122.601
F22	7.692	118.462	7.68	118.756	7.675	118.648
Q23			8.897	123.8	8.902	123.99
A24	8.864	126.657	8.807	126.024	8.795	126.098
E25			7.73	113.66	7.939	114.121
I26	7.285	120.163	7.333	121.327	7.317	121.286
A27	8.209	121.386				
Q28	7.957	118.521	7.691	118.611	7.768	118.764
L29	8.145	123.169	8.348	123.745	8.28	123.79
M30	8.351	116.786			8.37	116.971
S31	7.628	111.75	7.527	111.511	7.566	111.418
L32	7.828	121.955	7.764	122.005	7.8	122.021
I33	8.17	119.936	8.33	119.301		
I34	8.127	116.542	8.112	116.302	8.085	116.07
N35	7.716	114.484	7.663	115.009	7.741	114.777
T36	7.318	116.887	7.323	117.059	7.319	117.063
F37			8.422	125.115	8.429	125.196
Y38			6.879	123.736	7.008	124.113
S39			8.579	116.877	8.605	117.102
N40			8.337	119.668	8.403	120.034
K41	7.906	117.102	7.854	116.279	7.827	116.128
E42	8.408	114.367	8.346	114.093	8.383	114.017
I43	6.821	112.217	6.828	112.082	6.756	111.563
F44	7.984	120.641	7.963	120.448		
L45	5.585	122.512	5.639	122.745	5.609	122.871
R46	6.452	117.348	6.486	117.685	6.433	117.688
E47	7.712	116.827	7.633	116.527	7.634	116.518
L48	7.877	119.417				
I49	8.237	120.125				
S50	8.26	116.819	8.287	116.486	8.307	116.291
N51	7.895	120.423	7.232	119.511	7.223	119.42
S52	8.386	120.701				
S53	8.582	115.934				
D54	8.2	119.436	8.557	119.671	8.534	119.896

A55	7.841	123.048	7.674	122.727	7.637	122.7
L56	8.484	124.553	8.408	125.391	8.458	125.464
D57	8.83	119.551	8.793	119.503	8.796	119.581
K58	7.765	117.314	7.751	117.274	7.719	117.218
I59	7.361	116.99	7.428	117.264	7.436	117.247
R60	8.589	126.91	8.571	126.287	8.586	126.653
Y61	8.686	118.401	8.67	118.746	8.669	118.753
E62	8.097	119.288	8.159	119.354	8.16	119.366
S63	8.318	114.15	8.29	114.22	8.291	114.224
L64	7.378	121.709	7.385	121.691	7.384	121.686
T65	7	106.528	6.995	106.559	6.994	106.59
D66	7.312	118.138	7.326	118.001	7.332	118.029
P67						
S68	8.065	115.202	8.063	115.149	8.069	115.139
K69	7.85	123.257	7.876	123.331	7.877	123.344
L70	7.223	111.7	7.227	111.497	7.229	111.505
D71	8.025	123.384	8.045	123.629	8.042	123.622
S72	7.462	109.077	7.479	109.074	7.48	109.064
G73	7.66	111.443	7.657	111.424	7.654	111.409
K74	8.924	127.139	8.919	127.039	8.923	127.108
E75	7.637	116.571	7.63	116.709	7.627	116.777
L76	8.67	127.263	8.703	127.186	8.696	127.256
H77	7.666	118.845	7.666	118.486	7.69	118.703
I78	8.178	117.582	8.16	117.686	8.148	117.613
N79	9.742	125.169	9.754	125.135	9.763	125.171
L80	9.026	121.965	9.046	121.999	9.044	121.975
I81	9.354	120.075	9.346	120.063	9.339	120.056
P82						
N83	9.154	121.469	9.17	121.723	9.168	121.695
K84	9.468	124.919	9.458	124.905	9.458	124.9
Q85	8.472	119.664	8.466	119.709	8.496	119.817
D86	7.677	116.458	7.708	116.311	7.692	116.342
R87	7.903	120.713	7.902	120.579	7.901	120.557
T88	8.054	106.115	8.058	106.216	8.056	106.176
L89	8.43	122.372				
T90	8.035	123.935	8.021	123.805		
I91	9.456	127.683	9.419	127.828	9.403	127.807
V92	9.649	129.661	9.685	129.847	9.682	129.859
D93	9.482	123.327	9.583	123.391	9.576	123.398
T94	7.257	108.962	7.196	108.716	7.204	108.591
G95	9.556	108.742	9.924	109.256	9.924	109.271
I96	6.862	115.685	6.883	115.869	6.874	115.797
G97	7.687	103.44	7.92	103.141	7.915	103.028
M98	7.686	116.778	7.821	116.698	7.791	116.698

T99	8.051	112.145	8.011	111.799	7.96	111.84
K100	8.622	121.245				
A101	7.899	116.498	7.873	116.527	7.855	116.327
D102	7.425	117.387	7.466	117.264	7.482	117.315
L103						
I104	7.581	115.949	7.383	115.177	7.394	115.149
N105	8.304	117.365	8.427	116.451	8.443	116.833
N106	8.793	116.117	8.647	115.659	8.696	115.698
L107			7.162	120.59	7.193	121.004
G108			6.855	132.127	6.9	131.638
T109			7.512	107.318	7.583	107.012
I110			7.894	122.749	7.887	122.433
A111[a]			8.575	126.728	8.612	127.179
K112			8.682	122.088	8.661	122.497
S113						
G114	8.795	110.061			9.013	109.375
T115						
K116						
A117	7.922	120.579				
F118	8.341	120.067				
M119	7.863	117.299	7.886	117.18	7.854	117.246
E120	8.103	118.587	8.059	118.361	8.089	118.375
A121	7.797	124.045	7.877	123.806	7.826	123.997
L122	8.337	120.263			8.439	119.791
Q123	7.83	119.468	7.911	119.572	7.926	119.824
A124	7.4	120.172	7.431	120.19	7.402	120.154
G125	7.666	106.002	7.69	106.128	7.676	105.982
A126	8.118	123.077	8.126	123.222	8.137	123.24
D127	8.198	118.476	8.306	119.09	8.368	118.985
I128	7.723	123.117	7.837	124.861	7.77	124.53
S129	8.556	114.268	8.611	114.811	8.645	114.663
M130	7.995	120.138			8.114	119.432
I131	7.83	119.395			7.906	119.812
G132	7.725	109.069			7.145	107.147
Q133	7.993	120.204			7.95	119.505
F134	7.346	115.534			7.242	115.984
G135	7.651	105.585			7.961	107.53
V136	6.595	108.057			6.549	107.15
G137	8.746	107.562			7.186	104.598
F138	9.126	123.546	9.674	124.177	9.76	125.914
Y139	7.144	110.078			7.082	110.369
S140	7.999	114.248	7.857	114.273	7.897	114.15
A141	8.002	125.259			8.077	125.329
Y142	7.405	111.945	7.487	112.05	7.484	111.987

L143	8.06	118.517	8.081	118.721	8.06	118.461
V144	6.597	102.508	6.545	102.534	6.562	102.475
A145	7.691	124.185	7.647	124.315	7.722	124.309
E146	8.524	117.845	8.491	117.296	8.537	117.62
K147	7.416	116.914	7.38	117.043	7.414	117.092
V148	8.056	125.672	8.061	125.933	8.066	126.024
T149	9.087	124.768	8.996	124.641	9.046	124.659
V150	10.486	128.178	10.484	128.435	10.473	128.422
I151	9.63	128.498	9.54	128.387	9.531	128.27
T152	9.17	121.893	9.178	122.219	9.166	122.241
K153	8.903	127.159	9.106	127.567	9.1	127.645
H154	9.707	131.784			9.708	131.738
N155	9.647	125.919				
D156						
D157						
E158	9.348	117.604	9.271	117.447	9.294	117.407
Q159	8.15	119.433	8.125	119.285	8.139	119.414
Y160	8.682	124.265	8.693	124.444	8.685	124.41
A161	9.316	119.709	9.301	119.59	9.305	119.628
W162	10.38	130.215	10.279	130.434	10.29	130.54
E163	8.656	126.12	8.749	126.332	8.769	126.374
S164	8.262	113.321	8.329	113.697	8.351	113.707
S165			9.525	130.756		
A166	9.122	119.645	9.118	119.558	9.183	119.665
G167	8.487	106.773	8.496	106.497	8.501	106.688
G168	8.51	106.283	8.49	106.383	8.534	106.401
S169	7.794	115.569	7.791	115.516	7.786	115.62
F170	8.817	119.446	8.851	119.59	8.847	119.724
T171	8.791	109.185	8.824	109.228	8.844	109.198
V172	8.874	117.79	8.866	117.845	8.856	117.78
R173	9.109	124.067	9.086	124.159	9.092	124.125
T174	9.128	118.401	9.083	118.19	9.083	118.114
D175	8.652	126.346				
T176	8.043	115.77	8.046	115.67	8.041	115.636
G177	7.802	109.992	7.779	109.988	7.768	109.971
E178	8.282	120.812	8.283	120.865	8.279	120.835
P179						
M180	9.469	123.926	9.451	123.94	9.44	123.958
G181	8.628	111.4	8.636	111.191	8.63	111.125
R182	7.461	120.045	7.492	120.037	7.488	119.976
G183	9.202	115.684	9.227	115.797	9.235	115.754
T184	7.738	115.809	7.649	114.675	7.673	114.775
K185	10.039	128.204	10.039	127.375	10.032	127.319
V186	9.173	126.421	9.111	126.063	9.105	125.957

I187	9.989	127.677	9.974	127.729	9.968	127.685
L188	8.885	125.66				
H189	8.343	124.732				
L190	8.021	123.233	8.072	123.483		
K191	8.456	120.34	8.447	120.461		
E192	9.023	120.497	9.027	120.544	9.027	120.507
D193	8.382	113.123	8.365	113.098	8.384	113.146
Q194	7.863	118.707	7.872	118.473	7.87	118.488
T195	7.435	106.395	7.436	106.473	7.441	106.457
E196	8.872	123.8	8.891	123.828	8.885	123.765
Y197	6.644	114.509	6.672	114.508	6.665	114.495
L198	7.281	111.091	7.297	110.916	7.301	110.917
E199	7.309	116.444	7.313	116.469	7.311	116.459
E200	9.126	126.51	9.12	126.427	9.124	126.443
R201	8.917	116.056	8.914	116.095	8.914	116.091
R202	6.762	119.292	6.764	119.285	6.761	119.28
I203	8.12	117.574	8.128	117.546	8.12	117.596
K204	8.433	116.542	8.397	116.328	8.411	116.439
E205	7.708	118.435	7.711	118.585	7.733	118.526
I206	7.894	121.008	7.902	120.926	7.92	120.953
V207	8.374	120.626	8.407	120.986	8.443	121.416
K208	7.874	118.861	7.833	118.374	7.83	118.367
K209	7.511	116.225	7.499	116.206	7.52	116.165
H210	7.837	113.171	7.814	112.975	7.825	112.771
S211	8.172	115.42	8.169	115.705	8.176	115.683
Q212	8.151	120.009	8.117	120.03	8.097	120.022
F213	8.172	115.358	8.172	115.384	8.143	115.328
I214	7.127	121.591	7.102	121.629	7.085	121.666
G215	9.259	115.82	9.23	115.67	9.274	115.802
Y216	6.751	117.611	6.733	117.604	6.734	117.608
P217						
I218	8.092	123.304	8.103	123.545	8.066	123.495
T219	8.742	122.741	8.723	122.76	8.725	122.731
L220	8.711	127.616	8.72	127.694	8.716	127.713
F221	8.825	127.2	8.797	127.085	8.796	127.04
V222	7.938	121.443	7.92	121.55	7.939	121.427
E223	8.562	125.506	8.553	125.553	8.55	125.627

[a] The lid region (residues A111-G135) is highlighted in grey.
